# HiPSC-derived cardiomyocyte to model Brugada syndrome: both asymptomatic and symptomatic mutation carriers reveal increased arrhythmogenicity

**DOI:** 10.1186/s12872-023-03234-7

**Published:** 2023-04-25

**Authors:** Kirsi Penttinen, Chandra Prajapati, Disheet Shah, Dhanesh Kattipparambil Rajan, Reeja Maria Cherian, Heikki Swan, Katriina Aalto-Setälä

**Affiliations:** 1 Faculty of Medicine and Health Technology and BioMediTech Institute, Tampere University, Tampere, 33520 Finland; 2 Heart Hospital, Tampere University Hospital, Tampere, 33520 Finland; 3 Helsinki University Hospital, Helsinki, 00290 Finland

**Keywords:** Brugada syndrome, Human-induced pluripotent stem cell derived cardiomyocyte, Sodium, SCN5A, Arrhythmia

## Abstract

Brugada syndrome is an inherited cardiac arrhythmia disorder that is mainly associated with mutations of the cardiac voltage-gated sodium channel alpha subunit 5 (*SCN5A*) gene. The clinical symptoms include ventricular fibrillation and an increased risk of sudden cardiac death. Human-induced pluripotent stem cell (hiPSC) lines were derived from symptomatic and asymptomatic individuals carrying the R1913C mutation in the *SCN5A* gene. The present work aimed to observe the phenotype-specific differences in hiPSC-derived cardiomyocytes (CMs) obtained from symptomatic and asymptomatic mutation carriers. In this study, CM electrophysiological properties, beating abilities and calcium parameters were measured. Mutant CMs exhibited higher average sodium current densities than healthy CMs, but the differences were not statistically significant. Action potential durations were significantly shorter in CMs from the symptomatic individual, and a spike-and-dome morphology of action potential was exclusively observed in CMs from the symptomatic individual. More arrhythmias occurred in mutant CMs at single cell and cell aggregate levels compared with those observed in wild-type CMs. Moreover, there were no major differences in ionic currents or intracellular calcium dynamics between the CMs of asymptomatic and symptomatic individuals after the administration of adrenaline and flecainide.

In conclusion, mutant CMs were more prone to arrhythmia than healthy CMs but did not explain why only one of the mutation carriers was symptomatic.

**Supplementary Information:**

The online version contains supplementary material available at 10.1186/s12872-023-03234-7.

## Introduction

Brugada syndrome is an inherited cardiac arrhythmia disorder predisposing patients to ventricular tachycardia or fibrillation and sudden cardiac death. The diagnosis of Brugada syndrome mainly relies on specific ST elevation on surface electrocardiogram (ECG), history of ventricular tachycardia or fibrillation, family history of sudden cardiac death, and syncope occurrence [1, 2]. So far, the only effective treatment is the implantation of a cardioverter defibrillator [3], and the limited understanding of the cellular mechanisms underlying Brugada syndrome complicates the development of diagnostic and therapeutic approaches.

Brugada syndrome has been associated with mutations in 23 different genes [4]. Most mutations are in the voltage-gated sodium (Na^+^) channel alpha subunit 5 (*SCN5A*) gene coding for the alpha subunit of the cardiac Na^+^ channel (Na_V_1.5) responsible for the Na^+^ inward current (I_Na_) [5]. Loss- or gain-of-function mutations in SCN5A result in the dysfunction of Nav1.5, which leads to increased or decreased I_Na_ [6]. However, the link between a mutation and a disorder is rarely direct, and mutations in several genes can have a single clinical outcome, such as the Brugada syndrome [7]. Moreover, the clinical phenotype might differ even among carriers of the same mutation within one family, but underlying reasons for this phenotypic variability and incomplete penetrance are not always clear [7]. The human cellular phenotype of Brugada syndrome is not fully characterized because the available heterologous expression models lack many characteristics of native cardiomyocytes (CMs) and fail to fully recapitulate the disease [8]. Recently, the cellular mechanisms of Brugada syndrome have been investigated using human-induced pluripotent stem cell (hiPSC)-derived CMs (hiPSC-CMs) obtained from patients [9–17].

The hiPSC technology [18] is a valuable tool for studying genetic diseases and phenotypic variability. Here hiPSC lines were derived from two stepbrothers carrying a mutation in the *SCN5A* gene. One brother had been diagnosed with Brugada syndrome. The other one was asymptomatic, and arrhythmias could not be provoked by electrophysiological or pharmacological stimuli. We aimed to determine whether hiPSC-derived CMs (hiPSC-CMs) obtained from both stepbrothers presented different cellular phenotypes from one another and from that of healthy wild-type (WT) hiPSC-CMs.

## Materials and methods

### Clinical information

**Fig. 1:**
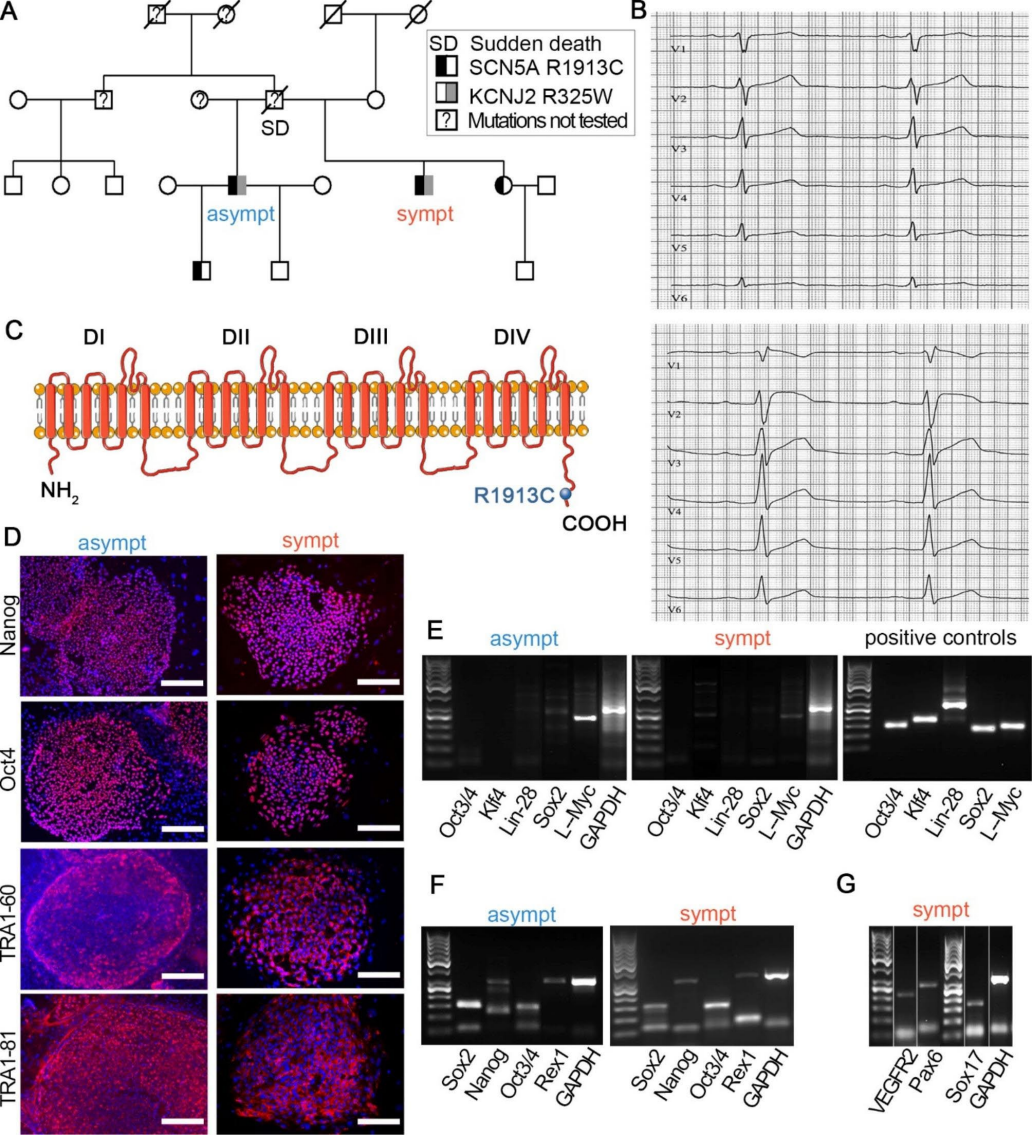
Clinical background of asymptomatic and symptomatic individuals and generation and characterization of hiPSC-lines. **(A**) The pedigree of the family of asymptomatic (Brugada syndrome) and symptomatic brothers. (**B**) ECG from symptomatic individuals before (above) and 10 min after receiving flecainide (below). (**C**) Schematic representation of *SCN5A* channel protein. Mutation R1913C is located in the C-terminal of the protein. (**D**) Immunocytochemical stainings and expression of pluripotency markers. Scale bars 100 μm. (**E**) RT-PCR confirmed that none of the exogenous genes are expressed in iPSC lines. Images are cropped and merged, and full images available in additional file 1: Fig S3A (**F**) pluripotency markers are turned on. Images are cropped and merged, and full images available in additional file 1: Fig S3B-C and (**G**) EBs express markers from all the three embryonic germ layers. Images are cropped and merged, and full images available in additional file 1: Fig S3D. GAPDH serves as a housekeeping gene in all RT-PRC figures

The pedigree of the family is presented in Fig. 1A. The symptomatic patient is a male diagnosed with Brugada syndrome and he carries the SCN5A-p.R1913C variant. This is likely pathogenic variant (referred later as mutation), since it is very rare in general population, in silico models predict it to be deleterious, mutation in the same codon has been reported to pathogenic and three paralogue pathogenic mutants are known. He was 32 years old at the time of the skin biopsy. He lost consciousness for the first time at home at night while having high fever in 2005. A prominent convex ST-segment elevation was observed in V1–V4. His next syncopal spell occurred in 2013 at an ice hockey rink. Later that year, he was resuscitated from an episode of ventricular fibrillation on the street. The ECG revealed slightly concave ST-segment elevation in all chests leads (up to 2 mm in V3). A typical example of spontaneous Brugada type 1 ECG from the symptomatic individual after collapse is presented in additional file 1: Fig S1. The patient’s cardiac ultrasound, magnetic resonance imaging, and coronary angiogram were always normal. An early repolarization and ST elevation in V1 and V2 (Fig. 1B) were observed in the flecainide test. Consequently, a cardioverter defibrillator was implanted. This patient also carries a R325W variant in the gene coding for the potassium inwardly rectifying channel subfamily J member 2 (KCNJ2-R325W). It has been associated with Andersen–Tawil syndrome, but an exercise stress test revealed no ventricular arrhythmias characteristic of Andersen–Tawil syndrome. However, the significance of the R325W variant in KCNJ2 is not known and with current knowledge, the pathogenicity of the variant cannot be verified or ruled out and thus it is a variant of unknown significance. Our patient did not have a history of periodic paralysis, and the resting ECG revealed no prominent U waves. The stepbrother of this patient presented both SCN5A-p.R1913C mutation and KCNJ2-R325W variant of unknown significance. He was 50 years old at the time of the skin biopsy. This individual never had cardiac symptoms, and the flecainide test revealed no sign of Brugada ECG or QT interval prolongation. The father from both stepbrothers had suddenly died at the age of 53.

### Generation and characterization of hiPSC lines

The hiPSC study was approved by the ethical committee of the Pirkanmaa Hospital District (R08070), and written informed consent was obtained from the participants. Skin fibroblasts were used to generate hiPSC lines. All the experiments were performed in accordance with relevant guidelines and regulations. The healthy control hiPSC line UTA.04511.WT was generated using Sendai vectors, and the healthy control line UTA.04602.WT was generated using pMX retroviral vectors without Cre-LoxP site, as described earlier [19, 20]. To generate hiPSC lines from the symptomatic (UTA.14,004.SCN5A) and asymptomatic (UTA.13,901.SCN5A) individuals, fibroblasts were transfected with the 4D-Nucleofector™ System (Lonza, Basel, Switzerland) according to the manufacturer’s protocols and using a plasmid DNA cocktail, as described previously [21]. Plasmid vectors are available from Addgene repository upon request: 27,077 (pCXLE-hOCT3/4-shp53-F), 27,078 (pCXLE-hSK), 27,080 (pCXLE-hUL), and 37,624 (pCXWB-EBNA1). After derivation, hiPSC lines were cultured on mouse embryonic fibroblast feeder cell layers (Merk Millipore, Darmstadt, Germany) as described previously [20]. One line from each subject was used.

The karyotypes, pluripotency, and embryoid body (EB) formation of all hiPSC lines were characterized. The characteristics of the WT hiPSC lines have been reported previously [19, 20]. Endogenous and exogenous gene expressions were examined *via* reverse transcription-polymerase chain reaction (RT-PCR). Detailed reaction conditions and PCR primers for the hiPSC characterization have been published previously [19]. The protein levels of endogenous pluripotency markers were studied *via* immunocytochemistry using antibodies recognizing sex-determining region Y-box 2 (SOX2), the homeobox protein NANOG, tumor-related antigen (TRA)1–81 (all at 1:200, from Santa Cruz Biotechnology, Santa Cruz, CA, USA), and the POU transcription factor OCT3/4 (1:400, R&D Systems) as described previously [22]. Karyotypes of the cell lines were obtained *via* a KaryoLite™ BoBs™ assay (Perkin Elmer) based on the BACs-on-Beads™ technology (Molecular and Systems Immunology and Stem Cell Biology, Turku Centre for Biotechnology, University of Turku, Finland). The expression of markers characteristic from the ectoderm (paired box protein *Pax6*), endoderm (*SOX17*), and mesoderm (vascular endothelial growth factor receptor 2 *VEGF-R2*) development was determined in EBs as described previously [22].

### Cardiomyocyte differentiation and characterization

All hiPSC lines were differentiated into spontaneously beating CMs by coculturing them with murine visceral endoderm-like (END-2) cells (from Prof. Mummery, Hubrecht Institute, Utrecht, The Netherlands). Minimum of four differentiation batches were established from each hiPSC lines. For calcium (Ca^2+^) imaging and patch-clamp studies, minimum of 30 days old beating cell clusters were mechanically excised and treated with collagenase A (Roche Diagnostics, Basel, Switzerland) [23]. Single CMs were then plated on 12- and 5-mm round-glass coverslips coated with 0.1% gelatin. For microelectrode arrays (MEA) (Multichannel systems, Reutlingen, Germany), spontaneously beating cardiac aggregates were dissected and plated on 0.1% gelatin-coated 6-well MEA plates. Single CMs and cardiac aggregates were maintained in EB medium supplemented with 20% fetal bovine serum. For immunofluorescence staining, the CMs were fixed with 4% paraformaldehyde, and the following primary antibodies were used: anti-cardiac troponin T (1:1,500, Abcam, Cambridge, MA, USA), anti-human Na_V_1.5 (hNa_V_1.5, 1:200, Alomone Labs, Israel, ASC-005), and anti-SCN7A (1:200, Sigma-Aldrich, HPA004879).

### Allelic imbalance and quantitative RT-PCR (qRT-PCR)

To determine the SCN5A allelic imbalance in hiPSC-CMs obtained from the asymptomatic and symptomatic subjects, beating CM aggregates were lysed, and total RNAs were purified using Norgen’s Total RNA Purification Plus Kit (Norgen Biotek Corp., Ontario, Canada) according to the manufacturer’s protocols. RNAs were converted into cDNAs using the High-Capacity cDNA Reverse Transcription Kit (Thermo Fisher Scientific, Massachusetts, USA). Allelic discrimination between WT and mutant *SCN5A* alleles was performed *via* quantitative PCR (qPCR) using standard curves as described before [24, 25]. To generate *SCN5A* allelic standard curves, plasmids containing either WT *SCN5A* or mutant SCN5A-p.R1913C allele were generated by amplifying a 576-bp fragment of *SCN5A* containing the mutation site from the cDNA extracted from the UTA.04602.WT cell line. The WT allele fragment was cloned into pBluescript SK + plasmid (Addgene, Cambridge, MA, USA) using BamHI and NotI restriction enzymes (Thermo Fisher Scientific Inc.). The mutant *SCN5A* plasmid was generated from the WT plasmid using the QuikChange II Site-Directed Mutagenesis Kit (Agilent Technologies Inc., Santa Clara, CA, USA). The primers used for generating the plasmids are listed in additional file 1: Table S1. Both plasmid constructs were sequenced to verify the presence of the correct allele fragment. Plasmids carrying WT and mutant *SCN5A* alleles were combined at the ratios (WT/mutant) of 1/0, 8/1, 4/1, 2/1, 1/1, 1/2, 1/4, 1/8, and 0/1 for the qPCR reactions. For allelic discrimination, the primers and probes were designed by the Custom TaqMan® SNP Genotyping Assay service (Thermo Fisher Scientific). The qPCR analyses were conducted using a 7300 Real-Time PCR System (Applied Biosystems, Life Technologies Ltd.) to determine the C_t_ values for the different plasmid ratios and the hiPSC-CM cDNA samples. For the plasmid standard curves, the log2 values of WT/mutant plasmid ratios were plotted against the corresponding ΔC_t_ values, which were obtained by subtracting the C_t_ value measured for the WT *SCN5A* allele from the C_t_ value measured for the mutant allele. The ΔC_t_ values for hiPSC-CM samples were measured and plotted on the standard curve to determine the corresponding WT/mutant ratio. In standard curves, the ΔC_t_ values for plasmid ratios were expressed as means ± standard deviations (SDs) (n = 3).

RNA samples were collected and extracted from spontaneously beating WT hiPSC-CMs and hiPSC-CMs derived from the asymptomatic and symptomatic subjects using Norgen’s Total RNA Purification Plus Kit (Norgen Biotek Corp., Ontario, Canada) according to the manufacturer’s protocols. The High-Capacity cDNA Reverse Transcription Kit (Applied Biosystems) was used to transcribe 500–1000 ng of RNA into cDNA, and qPCR was performed following standard protocols on the Abi Prism 7300 instrument (Applied Biosystems). Samples were analyzed as triplicates. The expression levels of *cardiac troponin T2* (*TNNT2*), *SCN5A*, *sodium/calcium exchanger 1 precursor* (*SLC8A1*), *ryanodine receptor 2* (*RYR2*), *protein phosphatase 3 catalytic subunit alpha* (*PPP3CA*), and the endogenous control *glyceraldehyde-3-phosphate dehydrogenase* (*GAPDH*) were evaluated using TaqMan Universal PCR Master Mix (Applied Biosystems). The following TaqMan assays (×20) were used: Hs00165960_m1 for *TNNT2*, Hs00165693_m1 for *SCN5A*, Hs01062258_m1 for *SLC8A1*, Hs00892883_m1 for *RYR2*, Hs00174223_m1 for *PPP3CA*,, and Hs00270914_m1 for *GAPDH* (Applied Biosystems). The relative expression levels were determined using the comparative method (ΔΔCt) [26]. The gene expressions were normalized to that of *TNNT2*.

### Action potential (AP) measurement and video recording

APs were recorded from spontaneously beating hiPSC-CMs in perforated patch configuration using amphotericin B at 36 °C ± 1 °C. Current-clamp recordings were digitally sampled at 20 kHz and filtered at 2 kHz using a low-pass Bessel filter on the recording amplifier. An Axon Series 200B patch-clamp amplifier connected to a Digidata 1440a AD/DA converter driven by the pCLAMP 11 software (Molecular Devices, California, USA) were used. The patch electrodes had tip resistance of 2.0–3.0 MΩ and contained the following intracellular solution: 132 mM KMeSO_4_, 4 mM ethylene glycol-bis (β-aminoethyl ether)-N,N,N′,N′-tetraacetic acid (EGTA), 20 mM KCl, 1 mM MgCl_2_, and 1 mM CaCl_2_ (pH was adjusted to 7.2 with KOH). The extracellular solution contained 143 mM NaCl, 4.8 mM KCl, 1.8 mM CaCl_2_, 1.2 mM MgCl_2_, 5 mM D-glucose, and 10 mM 4-(2-hydroxyethyl)-1-piperazineethanesulfonic acid (HEPES) (pH was adjusted to 7.4 with KOH). The AP durations at 90%/50% repolarization (APD90/APD50) ratio describe the shape of the repolarization curve and this ratio was used to classify the specific subtype of hiPSC-CMs. Only ventricular-like hiPSC-CMs, characterized by a ratio between AP durations at 90% and at 50% repolarization (APD90/APD50) < 1.35 (indicative of pronounced plateau phase) and an AP amplitude (APA) > 90 mV, were used for comparison. All the drugs and chemicals were purchased from Sigma-Aldrich (Sigma-Aldrich, Saint Louis, USA) unless otherwise specified. KMeSO_4_ was ordered from MP Biomedicals (California, USA).

Videos were simultaneously recorded to AP measurements using an ANDOR iXon 885 EM-CCD camera (Andor Technology, Belfast, Northern Ireland), TH4-200 light source (Olympus, Tokyo, Japan), inverted Olympus IX70 microscope with PhC 0.40NA 20× air objective (Olympus), and Live Acquisition software (TILL Photonics, Munich, Germany). Videos were recorded for at least 10 s at 60 frames per second. The pCLAMP software was configured to synchronize data between the patch-clamp and video recordings using synchronization pulses to determine the start and end of the video in the AP recording (additional file 1: Fig S2). From the recorded videos, half-width contraction and relaxation duration were calculated using a custom-made software based on MATLAB (The MathWorks, Massachusetts, USA) [27].

### Ionic current measurements

I_Na_ were recorded using whole-cell patch configuration at room temperature. For voltage-clamp experiments, patch pipettes were filled with a solution containing 140 mM cesium methane sulfonate, 5 mM NaCl, 5 mM EGTA, and 10 mM HEPES (pH adjusted to 7.2 with CsOH). The bath solution was composed of 50 mM NaCl, 115 mM tetramethylammonium chloride, 2 mM CaCl_2_, 1.2 mM MgCl_2_, 5 mM D-glucose, and 10 mM HEPES (pH adjusted to 7.4 with tetramethylammonium hydroxide). To block the Ca^2+^ current, 10-µM nimodipine was added to the extracellular solution. I_Na_ were elicited using step potentials ranging from − 60 to 80 mV with 5-mV increments and a holding potential of − 100 mV. The membrane capacitance was measured for each cell by integrating the area under the capacitive transient induced by a 5-mV hyperpolarizing step pulse, from − 80 to − 85 mV, and dividing it by the voltage step. Current densities were calculated by dividing the currents by the capacitance and were expressed as pA/pF. The liquid junction potential was not corrected. Series resistance and cell capacitance were compensated for 40–50%.

The Na^+^ conductance (g_Na_) was calculated using the equation g_Na_ = I_Na_/ (V_m_ − E_Na_), where V_m_ denotes the membrane potential and E_Na_ the Na^+^ reversal potential. The reversal potentials were experimentally determined for each cell. Steady-state voltage dependence of the inactivation was calculated using the double-pulse protocol with a holding potential of − 120 mV. The varying 200-ms-long conditioning pulse from − 120 to − 20 mV (∆V = 5 mV) protocol was used. The peak I_Na_ were normalized to a maximum value.

Both steady-state activation and inactivation curves were fitted using a Boltzmann equation: y = A/{1.0 + exp[(V_1/2_ –V)/k]}, where V_1/2_ denotes the half-maximum (in)activation potential and k the slope factor. Time constants of I_Na_ inactivation were determined by fitting a biexponential equation, y = A_f_ × [1–exp(–t/τ_f_)] + As ×[1–exp(–t/τ_s_)], where A_f_ and A_s_ are the fractions of the fast and slow inactivation components, respectively, and τ_f_ and τ_s_ are the time constants of the fast and slow inactivation components, respectively.

The time course of the recovery from inactivation was analyzed using the double-pulse protocol. For this, a conditioning pulse (P1) of − 20 mV was applied for 50 ms. Then, a test pulse (P2) of − 20 mV was applied for 50 ms after different recovery times (5, 10, 20, 50, 100, 200, 300, 400, 500, 600, 700, 800, 900, 1000, or 1500 ms) between each recovery potential. The peak I_Na_ elicited by P2 were normalized (P2/P1) and plotted as a function of the recovery interval. In addition, the time course of entry into the slow inactivation state was analyzed following the double-pulse protocol. For this, P1 at − 20 mV was applied for varying durations (5, 10, 20, 50, 100, 200, 300, 400, 500, 600, 700, 800, 900, 1000, 1500, or 2000 ms). They were followed by a step at–120 mV for 50 ms to remove fast inactivation and by P2 at − 20 mV for 50 ms to assess channel availability. The peak I_Na_ elicited by P2 were normalized (P2/P1) and plotted as a function of the duration of conditioning pulses.

The rapidly activating delayed rectifier potassium current (I_Kr_) was recorded in the presence of 5-µM nimodipine in the extracellular solution. I_Kr_ was sensitive to 1-µM E-4031 and elicited from the step potential from − 20 to 40 mV with 20-mV increments and a holding potential of − 40 mV. The peak and tail currents of I_Kr_ were calculated from the end of the test pulse and the peak of the tail current, respectively.

The Ca^2+^ currents were elicited from the step potential from − 50 to 60 mV with 5-mV increments and a holding potential of − 40 mV and were sensitive to 5-µM nimodipine. The time course of the recovery from inactivation was analyzed using the double-pulse protocol. For this, P1 of 10 mV was applied for 300 ms. Then, P2 of 10 mV was applied for 300 ms after different recovery times (10, 20, 40, 60, 80, 100, 150, 200, 300, 400, 600, 800, 1000, or 1200 ms) between each recovery potential. The peak I_Ca_ elicited by P2 were normalized (P2/P1) and plotted as a function of the recovery interval. The time course of the entry into the slow inactivation state was also analyzed following the double-pulse protocol. For this, P1 of 10 mV was applied for various durations (20, 40, 60, 80, 100, 200, 300, 400, 600, 800, 1000, 1200, 1500, or 2000 ms). It was followed by a 50-ms step at–40 mV to remove fast inactivation and by P2 at 10 mV for 300 ms to evaluate channel availability. The peak I_Ca_ elicited by P2 were normalized (P2/P1) and plotted as a function of the duration of conditioning pulses.

### MEA

MEA recording and analysis were conducted as described previously [28]. For the measurements, CM aggregates were moved into serum-free EB medium 1 h before the experiment. The measurements were performed in the serum-free EB medium at 36 °C ± 1 °C. The beating rate was corrected using Bazzet’s equation. Aggregates with a beating rate below 20 or above 90 were not considered to prevent over or under-correction.

### Ca^2+^ imaging

Dissociated spontaneously beating CMs were seeded on a coverslip and loaded with 4-µM Fluo-4 AM (Thermo Fisher Scientific). An HEPES-based medium, consisting of 137-mM NaCl, 5-mM KCl, 0.44-mM KH_2_PO_4_, 20-mM HEPES, 4.2-mM NaHCO_3_, 5-mM D-glucose, 2-mM CaCl_2_, 1.2-mM MgCl_2_, and 1-Na pyruvate (pH was adjusted to 7.4 with NaOH), was used for Ca^2+^ imaging measurements at 36 °C ± 1 °C. The Ca^2+^ kinetics were photographed using an Axio Observer.A1 microscope and an Objective Fluar 20x/0.75 M27 (both Carl Zeiss Microscopy GmbH, Göttingen, Germany). Images were obtained using an ANDOR iXon3 885 EM-CCD camera (Andor Technology, Belfast, Northern Ireland) synchronized with Lambda DG-4 Plus (Sutter Instrument, California, USA) wavelength switcher, ZEISS Filter set 69 (Carl Zeiss Microscopy GmbH, Göttingen, Germany), and ZEN 2 blue edition software (Carl Zeiss Microscopy GmbH, Göttingen, Germany). In addition to baseline measurements, the effects of 1-µM adrenaline (Sigma-Aldrich) and 1-µM flecainide (Sigma-Aldrich), used to induce clinical phenotypes in the hiPSC-CMs, were studied. For the Ca^2+^ analysis, the regions of interest were selected from spontaneously beating cells, and background noise was subtracted before data processing. All recordings lasted at least 30 s, with a sampling rate of 20 to 30 Hz.

### Data analysis and statistics

The electrophysiological and MEA data were analyzed using Clampfit version 11.1.0.23 (Molecular Devices) and a custom-made OriginLab program (OriginLab 2018b, Northampton, USA). For the patch-clamp and MEA recordings, comparisons between cell lines were performed with the non-parametric Kruskal–Wallis test followed by Dunn’s *post-hoc* multiple comparisons test (GraphPad Prism Version 5.02, San Diego, CA, USA). The Ca^2+^ peak parameters were analyzed using Clampfit (Molecular Devices). In these analyses, the instantaneous frequency was calculated by converting each interevent interval into a frequency and subsequently averaging these frequencies. In addition, Ca^2+^ traces were analyzed by identifying the signals as normal or abnormal and categorizing the abnormalities into subgroups. Data obtained from WT CMs (04511.WT and 04602.WT) were pooled as their Ca^2+^ peak parameter values were similar. Statistical analyses of Ca^2+^ and qRT-PCR data were conducted using the SPSS software version 24 (SPSS, Chicago, IL, USA). Comparisons within cell lines (before and after drug administration) were performed with non-parametric Wilcoxon and comparisons among cell lines (cell line-to-cell line comparison) with non-parametric Kruskal–Wallis with Bonferroni correction. Statistical significance levels are indicated as “ns” (not significant), * *P* < 0.05, ** *P* < 0.01, or *** *P* < 0.001.

## Results

### Analysis of hiPSC lines characteristics

We generated hiPSC lines from the skin biopsies of the asymptomatic and symptomatic stepbrothers. The SCN5A-p.R1913C mutation is caused by a single nucleotide change (5737 C > T) and is located in the C-terminus of the *SCN5A* gene (Fig. 1C). All hiPSC lines were characterized. WT hiPSC line characterization has been published before [19, 20, 29]. The hiPSC lines from the asymptomatic and symptomatic individuals expressed the endogenous pluripotent markers, namely, *NANOG*, *OCT4*, *TRA1-60*, and *TRA1-81* (Fig. 1D). The expression of exogenous reprogramming agents (*OCT3/4*, *Klf4*, *SOX2*, *LIN-28*, and *L-MYC*) was silenced (Fig. 1E), whereas that of endogenous pluripotency genes (*NANOG*, *REX1*, *OCT3/4*, and *SOX2*) was activated (Fig. 1F). Pluripotency of the cell lines was further confirmed by in vitro formation of EBs with all three germ layers (Fig. 1G). The karyotypes of all hiPSC lines were normal.

### Biochemical and genetic analyses of hiPSC-derived CMs

**Fig. 2:**
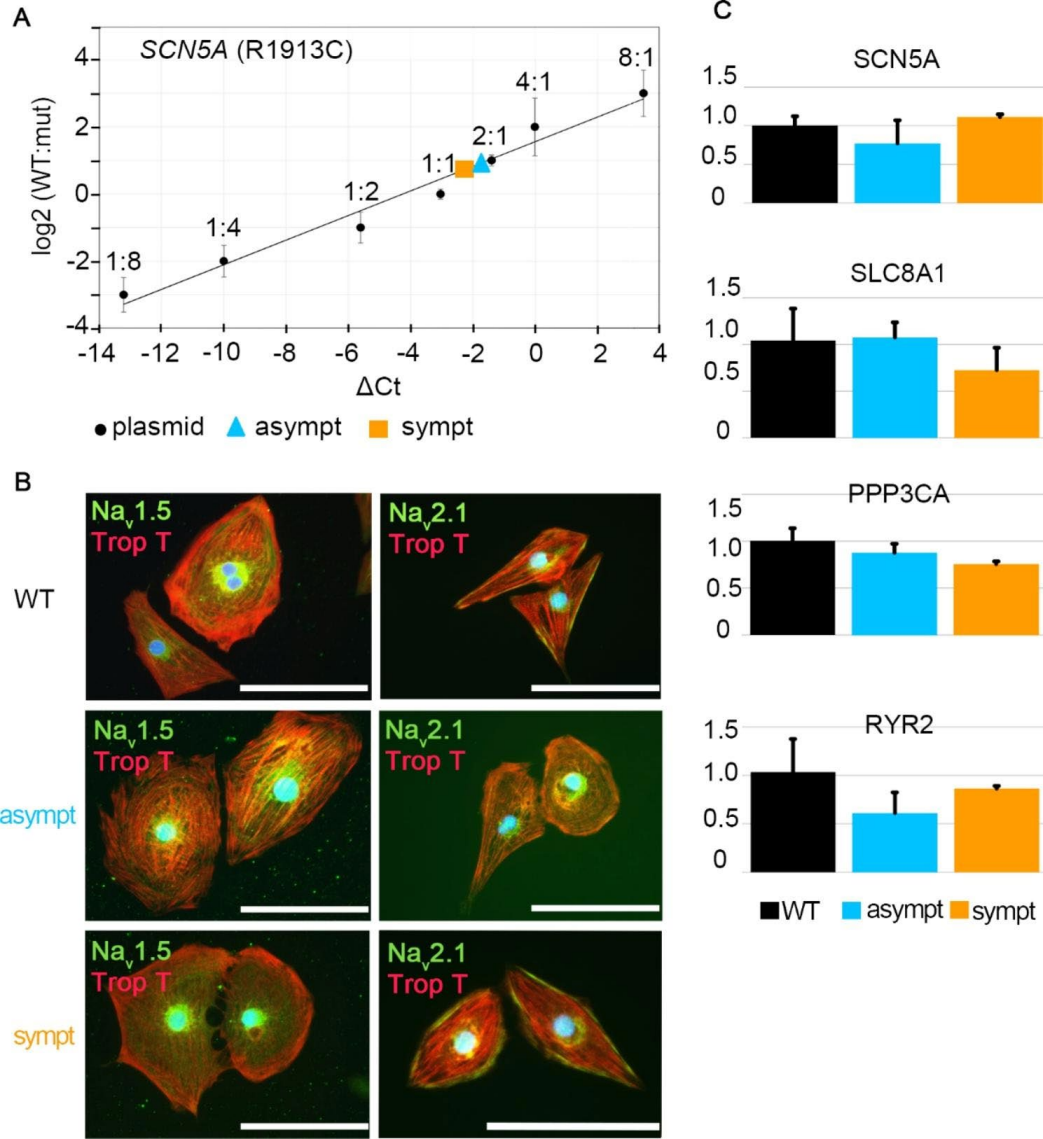
Characterization of hiPSC-CMs. (**A**) Allelic discriminations between the WT and mutated SCN5A alleles in asymptomatic and symptomatic CMs. (**B**) Immunocytochemical staining’s where red represents troponin T, green sodium channel Na_v_1.5 and Na_v_2.1 and blue DAPI-staining for nuclei. Scale bars 100 μm. (**C**) Relative gene expression levels of cardiac related genes. The GAPDH was used as an endogenous control. No statistically significant differences in expression levels of WT, asymptomatic and symptomatic CMs.

Allelic imbalance in CMs derived from both individuals was analyzed by discriminating between WT *SCN5A* and mutant SCN5A-p.R1913C alleles. The expression ratio between WT and mutant alleles was between 1/1 and 2/1 (WT/mutant) in cell lines obtained from both asymptomatic and symptomatic individuals (Fig. 2A).

Differentiated CMs expressed the cardiac protein marker troponin T and the Na^+^ channel proteins hNa_V_1.5 and hNa_v_2.1 (Fig. 2B). Immunofluorescence staining revealed no clear differences in the Na^+^ channel protein expressions between WT CMs and CMs from asymptomatic or symptomatic individuals by visual inspection. To confirm and compare the expression of certain ion channel genes, qRT-PCR analyses were conducted. No significant changes in *TNNT2*, *SCN5A*, *SLC8A1*, *RYR2*, and *PPP3CA*gene expression were observed among the three groups (Fig. 2C).

### Ionic currents and biophysics

**Fig. 3:**
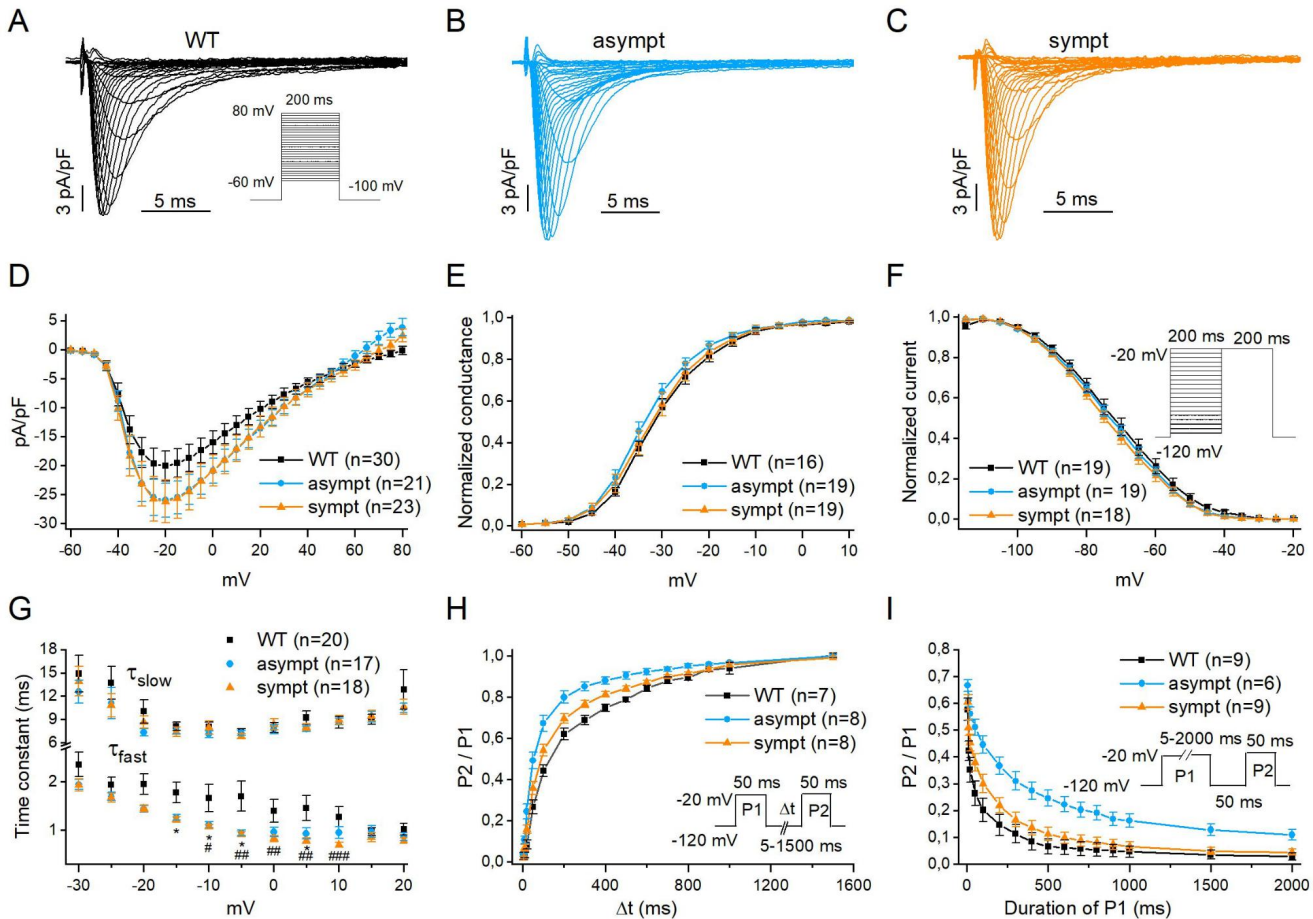
Sodium current (I_Na_) densities and gating properties of hiPSC-CMs. (**A-C**) Typical examples of whole-cell I_Na_ traces recorded at room temperature from WT, asymptomatic and symptomatic CMs. (**D**) Current-voltage relationships of average I_Na_ densities. (**E**) Average voltage dependence of activation. (**F**) Voltage-dependence of steady-state inactivation curve. Peak sodium currents were normalized to maximum values and plotted as function of voltage. (**G**) Average fast (τ_fast_) and slow (τ_slow_) time constants of I_Na_ inactivation plotted as a function of membrane potential. (WT vs. asympt and WT vs. sympt, *P < 0.05). (**H**) Time-course of recovery after inactivation. Peak I_Na_ elicited by P2 were normalized (P2/P1) and plotted as function of the recovery interval. (**I**) The time-course of entry into the slow inactivation state. Peak I_Na_ elicited by P2 were normalized (P2/P1) and plotted as function of the duration of P1. Insets: Voltage-clamp protocol used in each experiment. Numbers in parenthesis represent the number of cells used. Data are presented as mean ± S.E.M.

I_Na_ properties were studied in WT CMs (cell line UTA.04602.WT) and in CMs from asymptomatic and symptomatic individuals. Figure 3(A–C) present representative traces of I_Na_. The mean I_Na_ densities were plotted against the membrane potentials (Fig. 3D). Similar current–voltage relationships were found for all groups, and the maximum mean I_Na_ densities were observed at − 20 mV (Fig. 3D). Although averaged I_Na_ density at − 20 mV was approximately 25% greater in CMs from asymptomatic (–26.0 ± 2.9, n = 21) and symptomatic (–26.2 ± 3.7, n = 23) individuals than that in WT CMs (–20.1 ± 2.6, n = 30), there was no significance difference in I_Na_ densities among groups at any tested potentials (Fig. 3D, additional file 1: Table S2). Furthermore, the voltage dependence of I_Na_ activation was assessed from the current–voltage relationship of I_Na_ and plotted against the tested potentials (Fig. 3E). The V_1/2_ and k were calculated by fitting the curves with the Boltzmann distribution from WT CMs (V_1/2_ = − 31.0 ± 1.1, k = 6.0 ± 0.5; n = 16) and CMs from asymptomatic (V_1/2_ = − 33.5 ± 1.0, k = 5.4 ± 0.4; n = 19) and symptomatic (V_1/2_ = − 31.9 ± 1.7, k = 5.1 ± 0.4; n = 19) individuals. No significant difference was observed among the groups (S3A Table). I_Na_ inactivation was also investigated using the voltage protocol presented in Fig. 3F (inset). Similarly, V_1/2_ and k were calculated by fitting the curves with the Boltzmann distribution from WT CMs (V_1/2_ = − 71.5 ± 2.0, k = 9.9 ± 0.4; n = 19) and CMs from asymptomatic (V_1/2_ = − 73.1 ± 1.2, k = 10.5 ± 0.3; n = 19) and symptomatic (V_1/2_ = − 74.1 ± 1.5, k = 10.3 ± 0.3; n = 18) individuals. The values were similar among the groups (additional file 1: Table S3). To analyze the I_Na_ inactivation kinetics, a biexponential function was fitted to I_Na_ elicited at membrane potentials between − 30 and 20 mV in WT CMs (n = 20) and CMs from asymptomatic (n = 17) and symptomatic (n = 18) individuals. Then, τ_fast_ and τ_slow_ were calculated and plotted against the membrane potential. Our results indicated that τ_slow_ was similar among the groups, but τ_fast_ obtained with membrane potentials between − 15 and 10 mV were significantly smaller in CMs from the asymptomatic and/or symptomatic individuals than that in WT CMs (*P* < 0.05, Fig. 3G) (additional file 1: Table S4).

Additional experiments were conducted to characterize the differences in the time course of the recovery from inactivation using the double-pulse protocol presented in Fig. 3H (inset). These curves were fitted with biexponential equations. Data analysis revealed that τ_fast_ and τ_slow_ were smaller in CMs from asymptomatic (τ_fast_: 47.4 ± 4.9 and τ_slow_: 509.2 ± 60.5; n = 8) and symptomatic (τ_fast_: 63.3 ± 5.7 and τ_slow_: 579.9 ± 32.3; n = 8) individuals than those in WT CMs (τ_fast_: 74.8 ± 7.0 and τ_slow_: 700.3 ± 94.1). However, only the τ_fast_ difference between CMs from the asymptomatic individual and WT CMs was significant (*P* < 0.05) (additional file 1: Table S5A). A smaller τ_fast_, albeit not significant, was also observed in CMs from the symptomatic stepbrother compared with that of WT CMs (S5A Table). Furthermore, the time course of entry into the slow inactivation state was evaluated using the protocol presented in Fig. 3I (inset). These curves were also fitted with biexponential equations. The comparison revealed significantly greater τ_fast_ and τ_slow_ in the CMs from the asymptomatic individual than that in WT CMs (*P* < 0.05) (additional file 1: Table S5B).

The I_Kr_ current densities were not significantly different among the groups (additional file 1: FigS4A–B). The I_Ca_ current densities were similar between CMs from asymptomatic and symptomatic individuals, with the averaged I_Ca_ current densities being slightly, albeit not significantly, higher than that of WT CMs (additional file 1: FigS4C). The time course of recovery after inactivation and of entry into the slow inactivation state of I_Ca_ were also investigated, but neither τ_fast_ nor τ_slow_ were significantly different among the groups (additional file: Fig. S4D–G ).

### AP characteristics in CMs from asymptomatic and symptomatic individuals

**Fig. 4:**
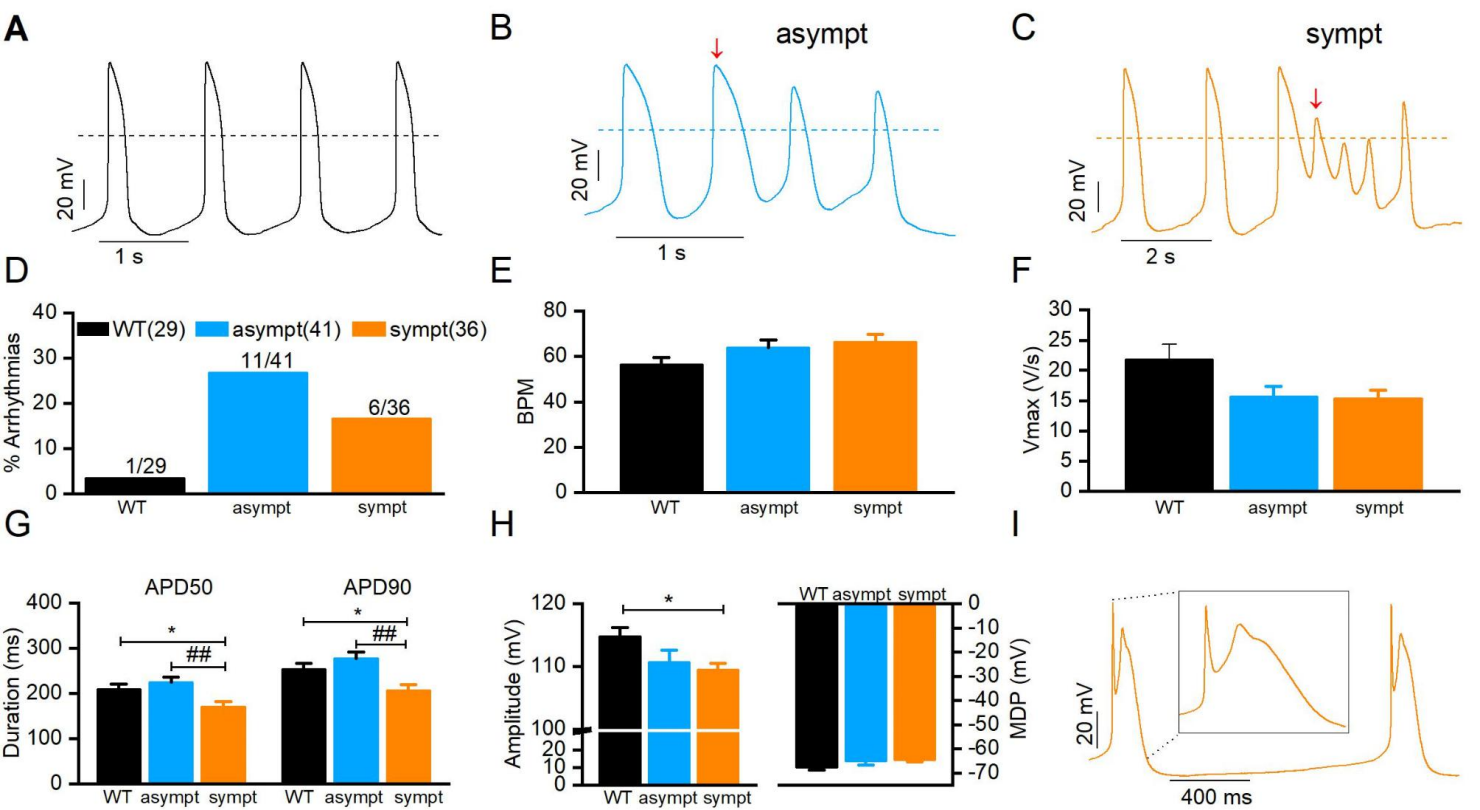
Action potential (AP) characteristics of ventricular-like CMs. Representative action AP traces from (**A**) WT, (**B**) asymptomatic and (**C**) symptomatic CMs respectively. Arrows indicate the arrhythmias in asymptomatic and symptomatic CMs. (**D**) Percentage of arrhythmia occurrences in WT (3.4%, n = 1/29), asymptomatic (n = 11/41) and symptomatic (n = 6/36) CMs. Averaged values and comparison of (**E**) beat per minute (BPM), (**F**) upstroke velocity (Vmax), (**G**) AP duration at 50% (APD50) and 90% (APD90) repolarization, and (**H**) AP amplitude (APA) and maximum diastolic potential (MDP). (**I**) Representative AP with prominent spike and dome morphology. Inset: enlarged version of AP. Numbers in parenthesis represent the number of cells used. * or # *P* < 0.05 * WT vs. asympt, # WT vs. sympt. Data are presented as mean ± S.E.M.

APs were recorded from spontaneously beating WT CMs (cell line UTA.04602.WT) and CMs from asymptomatic and symptomatic individuals. Figure 4 A–C presents representative AP traces. The occurrence of triggered activity (TA) in ventricular-like CMs was quantified and was greater in CMs from asymptomatic (26.8%, n = 11/41) and symptomatic (16.7%, n = 6/36) individuals than that in WT CMs (3.4%, n = 1/29) (Fig. 4D).

The beat per minute (BPM), maximal upstroke velocity (Vmax), APD_50_, APD_90_, APA, and maximum diastolic potential (MDP) of the APs were calculated. BPMs and Vmax were not significantly different among the groups (Fig. 4E, F). However, APD50 and APD90 were significantly shorter in CMs from the symptomatic individual (APD50: 170.5 ± 11.0 ms, APD90: 207.7 ± 12.4 ms, n = 36) compared with those in CMs from the asymptomatic individual (APD50: 224.6 ± 12.1 ms, APD90: 277.5 ± 14.5 ms, n = 41) and WT CMs (APD50: 209.9 ± 11.2 ms, APD90: 253.6 ± 13.1 ms, n = 29) (*P* < 0.05, Fig. 4G). APA was significantly smaller in CMs from the symptomatic individual (109.5 ± 1.0 mV, n = 36) than that in WT CMs (114.8 ± 1.4, n = 29) (*P* < 0.05, Fig. 4H left). The MDP was similar among the groups (Fig. 4H right). Interestingly, a prominent spike-and-dome morphology, which is the cellular equivalent of elevated ST segment observed in ECGs from patients with Brugada syndrome [30] was observed in AP recorded from CMs derived from the symptomatic individual only (Fig. 4I).

### Beating characteristics of CMs from asymptomatic and symptomatic individuals

**Fig. 5:**
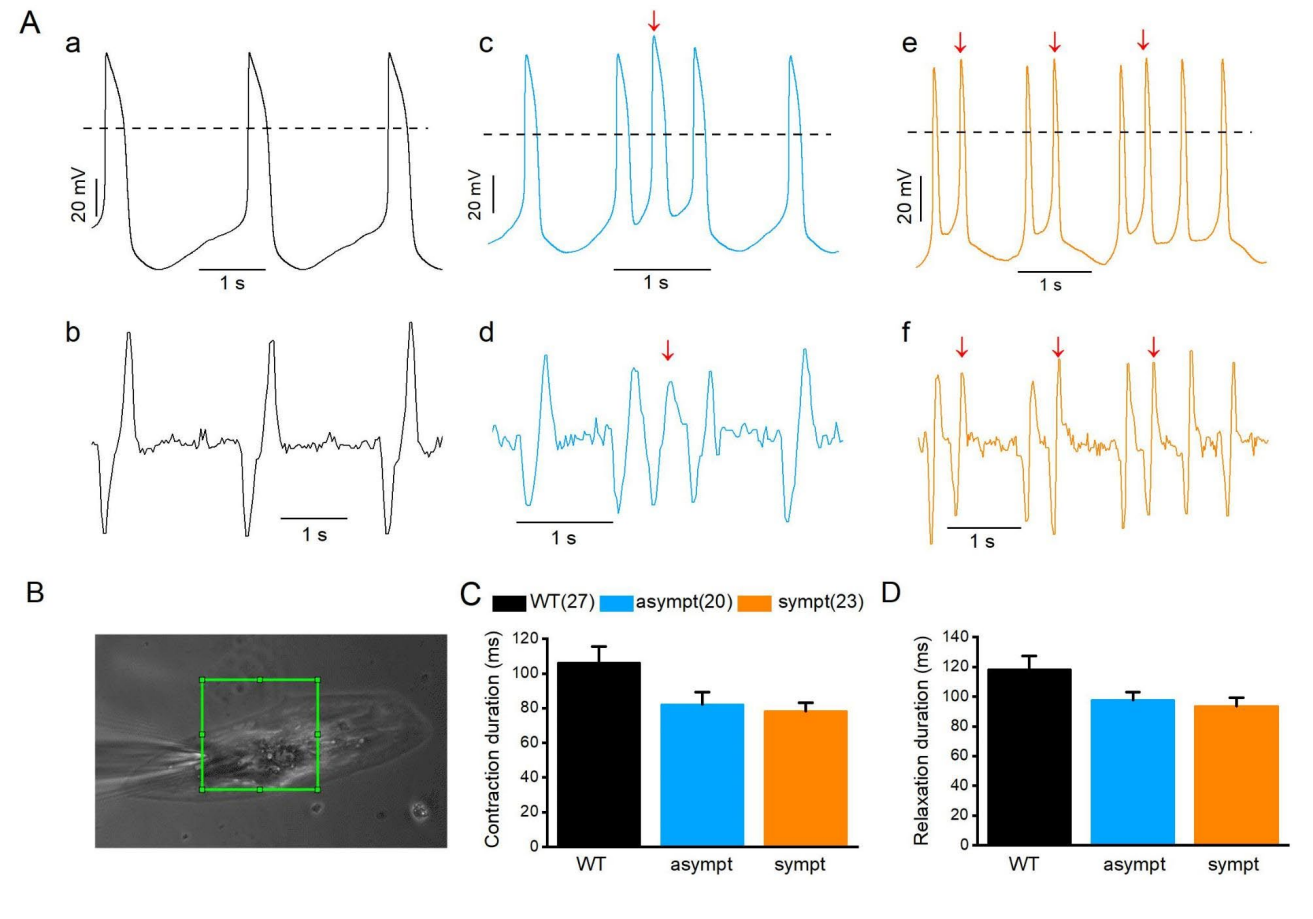
Simultaneous recording of action potential (AP) and contraction of same cells. (**A**) AP (upper panel) and corresponding beating traces (lower panel) recorded from same cells from WT (a,b), asymptomatic (c,d), and symptomatic (e,f) CMs. (**B**) Image of patch-clamp recording of CM where green rectangle represents the user-selected contraction region of interest. (**C-D**) Comparison of half-width contraction and relaxation durations measured from WT, asymptomatic, and symptomatic CMs. Numbers in parenthesis represent the number of cells used. Data are presented as mean ± S.E.M.

Videos of spontaneously beating WT CMs (n = 27) and CMs from asymptomatic (n = 20) and symptomatic (n = 23) subjects were recorded simultaneously in AP measurements. Figure 5A presents representative traces of normal AP (a) and the corresponding beating traces (b) recorded from WT CMs. TAs were also observed during simultaneous AP acquisition and video recording for CMs from asymptomatic (Fig. 5A c and d) and symptomatic (Fig. 5A e and f) individuals. These arrhythmic behaviors observed in APs (Fig. 5A c and e, marked in arrows) were also detected in the beating of CMs visualized by video recording (Fig. 5A d and f, marked in arrows). Figure 5B presents an image of patch-clamp recording of CMs and a user-selected region of interest for video analysis. Video analysis revealed that contraction and relaxation durations were shorter in CMs from asymptomatic (contraction: 82.3 ± 6.9 ms and relaxation: 97.6 ± 5.5 ms) and symptomatic (contraction: 78.3 ± 4.8 ms and relaxation: 93.8 ± 5.4 ms) individuals than those in WT CMs (contraction: 106.1 ± 9.4 ms and relaxation: 118.3 ± 9.2 ms), but the differences were not statistically significant (Fig. 5C–D and additional file 1: Table S6).

### Abnormal MEA measurements in CMs from asymptomatic and symptomatic individuals

**Fig. 6:**
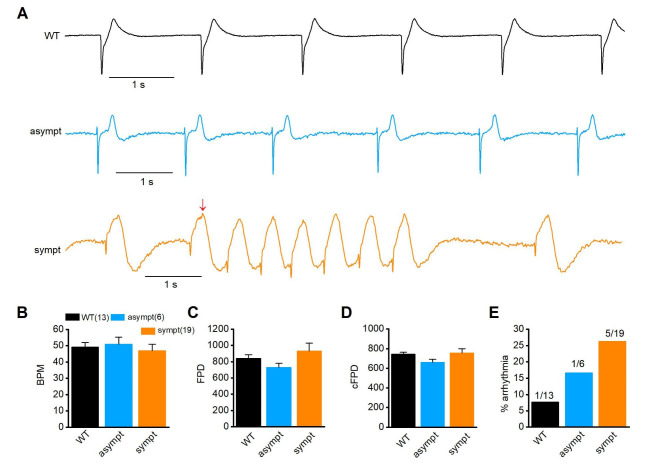
Characterization of hiPSC-CMs from MEA recording. (**A**) Representative Microelectrode array (MEA) recording from WT (a), asymptomatic (b) and symptomatic-CMs (c). Arrow indicates the initiation of triggered arrhythmia in symptomatic CMs. Comparison of beats per minute (**B**), field potential duration (FPD) (**C**) corrected FPD (**D**) and (**E**) occurrence of arrhythmias from WT, asymptomatic and symptomatic CMs. Numbers in parenthesis represent the number of cells used. Data are presented as mean ± S.E.M.

The electrophysiological properties of CM aggregates were also evaluated using MEA. Figure 6 A presents representative traces of MEA recording from WT CMs and CMs from the asymptomatic and symptomatic individuals. All CMs had similar beating rates and field potential durations (FPDs) (Fig. 6B, C and S6 Table). The FPDs were corrected with the corresponding beat rates using Bazzet’s equation. No significant differences in corrected FPDs were observed among groups (WT: 743.6 ± 21.7 ms, n = 13, asymptomatic: 659.4 ± 32.1 ms, n = 6, and symptomatic: 755.2 ± 42.5 ms, n = 19) (Fig. 6D and additional file 1: Table S7). Moreover, the percentage of arrhythmia in the MEA recording was higher for CMs from asymptomatic (16.7%, n = 6) and symptomatic (26.3%, n = 19) individuals than that from WT CMs (7.7%, n = 13) (Fig. 6E).

### Ca^2+^ abnormalities in CMs from asymptomatic and symptomatic individuals

**Fig. 7:**
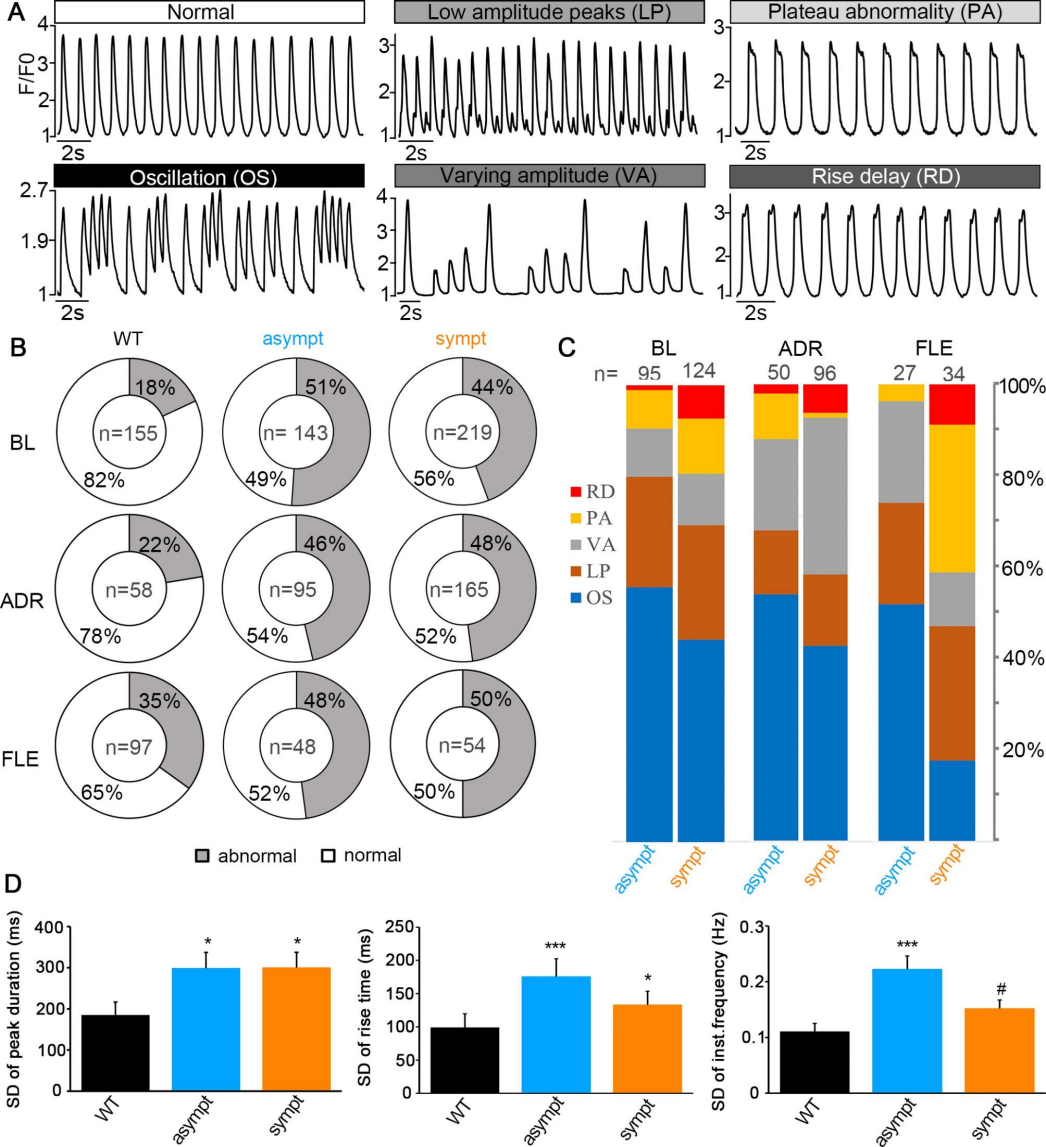
Ca^2+^ handling abnormalities in hiPSC-CMs. (**A**) Representative traces of a control CM showing normal regular Ca^2+^ transients and mutated CMs showing different abnormality types including oscillations (OS), low amplitude peaks (LP), varying amplitude (VA), plateau abnormality (PA) and rise delay (RD). (**B**) Doughnut charts indicating the abnormality percentage of WT, asymptomatic and symptomatic CMs in baseline and during adrenaline and flecainide perfusion. (**C**) Bar charts indicating the percentage of each abnormality type of asymptomatic and symptomatic CMs in baseline, and during adrenaline and flecainide perfusion. (**D**) Bar graph comparisons of standard deviation (SD) of peak duration, rise time 10–90% and instantaneous frequency of WT (n = 155), asymptomatic (n = 143) and symptomatic (n = 219) CMs in baseline. Data are presented as mean ± S.E.M. * or # indicates P < 0.05 and *** P < 0.001. * indicates comparisons with WT CMs and # indicates comparisons with asymptomatic CMs.

Abnormalities of the Ca^2+^ transients such as oscillations (OS), low amplitude peaks (LP), varying amplitude (VA), plateau abnormality (PA), and delay in the Ca^2+^ upstroke (rise delay, RD) (Fig. 7A) were observed in hiPSC-CMs. Abnormalities were defined as follows: (1) OS were detected if Ca^2+^ oscillated for two or more peaks without reaching the baseline, (2) LP were small amplitude Ca^2+^ events of at least 10% of the preceding Ca^2+^ spike amplitude, (3) PA was detected if the decay time of the Ca^2+^ upstroke was prolonged, (4) RD was detected if the rise time of the Ca^2+^ upstroke was prolonged, and (5) VA was detected if the amplitude of the peaks varied continuously. Transients were considered normal if the trace excluded any of these abnormalities.

More abnormalities of the baseline Ca^2+^ transients were observed in CMs derived from asymptomatic and symptomatic individuals than in WT CMs (51%, 44%, and 18% of the cells, respectively), but there was not much difference between the symptomatic and asymptomatic groups (Fig. 7B). The effects of adrenaline and flecainide were tested to induce the clinical phenotype of Brugada syndrome in CMs. Adrenaline did not impact the percentage of abnormalities in WT CMs or CMs from the asymptomatic or symptomatic individuals (22%, 46%, and 48% of the cells, respectively) compared with those measured without treatment (Fig. 7B). Flecainide increased the proportion of abnormalities in WT CMs (35%), but not in CMs from asymptomatic and symptomatic individuals (48% and 50% of the cells, respectively, Fig. 7B). In transients of untreated CMs from asymptomatic and symptomatic individuals, the percentages of the different abnormality types were similar, and mostly OS and LP were found. The main effect of adrenaline was the increase in the amount of VA in CMs from the symptomatic individual from 11 to 34% and from the asymptomatic subject from 11 to 20% (Fig. 7C). Flecainide induced an increase in the proportion of PA from 9 to 32% in CMs from the symptomatic individual and VA from 11 to 22% in CMs from the asymptomatic subject (Fig. 7C).

In the absence of treatment, averaged Ca^2+^ peak parameters (additional file 1: Table S8) were significantly different between WT and mutant CMs with lower Ca^2+^ transient amplitudes (F/F0), Ca^2+^ peak durations (Ca^90^), half-widths, 90–10% decay times, and interevent intervals and higher event frequencies in CM from asymptomatic and symptomatic individuals. In addition, the event frequency was significantly higher in CMs from the asymptomatic individual than that in CMs from the symptomatic subject (additional file 1: Table S8). The variation (SD) of Ca^90^ and 10–90% rise time were significantly higher in CMs from asymptomatic and symptomatic subjects than those in WT CMs (Fig. 7D), indicating an abnormal beating pattern of mutated cells. Significantly more variations of the instantaneous frequency were observed in CMs from the asymptomatic individual than in WT CMs and CMs from the symptomatic subject (Fig. 7D).

Adrenaline affected all CMs in a similar way by significantly decreasing F/F0, Ca^90^, half-width, 90–10% decay time, and interevent interval and by increasing the event frequency (additional file 1: Table S8). Cytosolic Ca^2+^ transient amplitudes during adrenaline perfusion were significantly lower in CMs from the symptomatic individual than in WT CMs (additional file 1: Table S8). In addition, both types of mutant CMs had significantly lower Ca^90^, half-width, 90–10% decay time, and interevent interval and higher event frequency during adrenaline perfusion than those in WT CMs (additional file 1: Table S8). In addition, the interevent intervals were significantly smaller and the event frequency significantly higher in CMs from the asymptomatic subject than those in CMs from the symptomatic individual.

Flecainide affected all CMs in a similar fashion by significantly decreasing cytosolic Ca^2+^ transient amplitudes and event frequencies and by increasing Ca^90^, half-width, and interevent interval (additional file 1: Table S9). No significant differences were observed in the Ca^2+^ peak parameters among CMs in the absence or presence of flecainide.

## Discussion

In this study, we successfully generated hiPSC-CMs from two individuals carrying an SCN5A-p.R1913C mutation. Only one of them showed clinical signs of Brugada syndrome including typical ECG findings, whereas the other one was clinically asymptomatic, and no ECG changes could be provoked.

The mutant and WT alleles were equally expressed in CMs from asymptomatic and symptomatic individuals and did not account for the different clinical phenotypes of the individuals. A balanced allele expression was also been reported in a previous study on Brugada syndrome using hiPSC-CMs with the *SCN5A* mutation p.S1812X [14]. In the present work, the Na^+^ channel protein and gene expression levels were not significantly different among the symptomatic and asymptomatic SCN5A-p.R1913C mutation carrier CMs and WT CMs. From the voltage-clamp experiments, the I_Na_ were similar in mutant CMs, with a tendency to be higher than that of WT CMs. Similarly, in a Finnish family with an SCN5A-p.I141V mutation, I_Na_ was higher, albeit not significantly, in mutant CMs [31]. Other studies reported a decrease in I_Na_ in hiPSC-CMs obtained from patients with Brugada syndrome and demonstrated a reduced Vmax in CMs derived from patients compared with that in CMs obtained from healthy subjects [9, 12–15]. Although Vmax is often used as an I_Na_ indicator [32], a nonlinear relationship between Vmax and I_Na_ has also been reported [33, 34]. Furthermore, a lower Vmax in hiPSC-CMs does not always ensure a lower I_Na_, as CMs might have more positive MDP at which a fraction of Na^+^ channels gets inactivated and is thus not functionally available [35]. Our results showed that the SCN5A-p.R1913C mutation results into speeding up the onset of inactivation particular at the voltages where activation of sodium channel reach to the maximum level. Early studies also showed that mutation in sodium channel causing Brugada syndrome result into faster inactivation of the sodium channel [36, 37]. The SCN5A-p.R1913C mutation caused faster inactivation in both symptomatic and asymptomatic hiPSC-CMs. Therefore, the Brugada phenotype cannot be explained solely by inactivation defect. Here, the SCN5A-p.R1913C mutation caused a faster recovery from the inactivation of Na^+^ channels. Faster recovery from the inactivation has been shown to result in increased I_Na_ [6]. Furthermore, another study on Brugada syndrome using hiPSC-CMs harboring the SCN5A-p.R367H mutation revealed a faster recovery from inactivation in mutant CMs [11], which is one of the underlying causes of arrhythmia occurrence in patients with Brugada syndrome [38]. In the present work, a higher degree of arrhythmias was observed in AP, intracellular Ca^2+^, and MEA recordings in CMs from asymptomatic and symptomatic individuals compared with that of WT CMs. There are various mechanisms for the occurrence of the triggered arrhythmias. Earlier study suggest that phase 3 early after depolarization can occur in shorter AP duration as the result of activation of sodium calcium exchanger by larger intracellular calcium at the end of AP [39]. The AP arrhythmias were also reflected in the beating behavior observed on video, thus showing the dependency between the electrical and mechanical activities of CMs. Reduced Ca^2+^ transient amplitudes might lead to insufficient Ca^2+^ release from the sarcoplasmic reticulum and cause an alteration in the Ca^2+^ removal *via* the sarcoplasmic reticulum Ca^2+^-ATPase and Na^+^/Ca^2+^ exchanger [9]. This might lead to the decreased decay time detected in mutant CMs. Some of the Ca^2+^ cycling pathways might be affected in the CMs from patients with Brugada syndrome. The increased arrhythmia might be attributed to increased variations in the beat rate and other parameters, as previously reported [9, 40, 41]. The effects of adrenaline and flecainide were investigated to determine whether the Brugada phenotype could be induced. However, no such phenotypic behavior was observed in Ca^2+^ peak parameters in mutant and WT CMs, although adrenaline induced significant changes in mutant CMs.

The APD in CMs from the symptomatic subject was shorter than that in WT CMs and CMs from the asymptomatic individual. The AP shape and APD depend on the fine-tuning of inward and outward ionic currents and their interactions [42]. To determine the cause of shorter APD in CMs from the symptomatic subject, we investigated the I_Kr_ and I_Ca_ current densities in WT CMs and mutant CMs. No differences were observed between the mutant lines. In addition, APD also depend on the BPM and hiPSC-CMs from symptomatic individual had the highest BMP, which partially explain the shortest APD. However, the mechanism behind the difference in APD was not fully explained. Other ionic currents, such as the late sodium current, slowly activating delayed rectifier potassium current, transient outward potassium current, inward rectifier potassium current, and Na^+^/Ca^2+^ exchanger, also influence the APD [42]. Brugada syndrome is characterized by an ST-segment elevation in the right precordial leads of a 12-lead ECG accompanied by a J wave [30, 43]. The I_to_-mediated AP notch and loss of the dome mainly in the right ventricle epicardium, but not in the endocardium, cause the transmural voltage gradient during ventricular repolarization and, consequently, the characteristic ECG changes [30]. Here, the spike-and-dome morphology was only observed in the AP of CMs from the symptomatic subject. An early study demonstrated that the spike-and-dome morphology in APs coincides with the J waves in the ECG [43]. Although the mutations occur in all Na^+^ channels in the heart, they often manifest only in one chamber, especially the right ventricle. In addition, flecainide inhibits I_Na_ in all CMs, but the Brugada phenotype is particularly observed in the right ventricle [44]. This might be because ionic currents and biophysics of ion channels differ not only between chambers but also between the left and right parts of one chamber [44, 45]. Thus, cardiac diseases should ideally be studied using specific types of CMs. However, it is not possible to obtain region-specific CMs from hiPSCs with high purity, and only a small portion of epicardiac-like CMs is yielded using the END-2 differentiation method [46], which hinder the detailed investigation of mechanisms inducing the Brugada phenotype in hiPSC-CMs.

Polygenic risk by common variations in the genetic background and modifiers might cause phenotype differences. Some of these variations might affect cardiac-specific proteins and Na^+^ current’s biophysical properties, thus inducing different clinical phenotypes among carriers of the same mutation. The link between a mutation and a clinical disease is rarely direct [47]. An individual with Brugada syndrome typically begins exhibiting symptoms around the middle age (average age on diagnostic: 45 years) [48]. Asymptomatic and symptomatic CMs did not fully recapitulate the clinical phenotype of Brugada syndrome possibly due to the inefficient recapitulation of late-onset disease and the lack of maturity of hiPSC-derived cells [49].

The pathophysiology of Brugada syndrome includes various factors beyond the mutation in sodium channels and genetic background and modifiers play an important role [50]. The different genetic backgrounds confer disparate susceptibilities to the effect of mutant sodium channel [7]. Studies showed that only ∼50% of the genome is shared between siblings and phenotypic differences could be the result of DNA variants in the other ∼50% of the genome rather than the disease-associated mutations [51]. Thus, different genetic background from two different mothers of two patients might have an effect in the results. Another limitation in this study is that the healthy individual is not from the same family. Adding one more control from the same family would have strengthen our results. An ideal control for this study would be the genome edited hiPSC-CMs with same genetic background and difference is only the absence of mutation [51]. However, genome editing of this mutation is left for the further studies. In addition, we had some technical limitation also existed in this study. The frame rate of the video recording was limited and video recording at higher frame rate could be able to record smaller change in membrane potential. Furthermore, we only use one iPSC line from all individuals. Addition of one more clone line from all individuals might strengthen our results. Furthermore, the limitation of immaturity of hiPSC-CMs should also be considered. It might cause different expression profiles of ion channels, lower Vmax and I_Na_, which might explain the lack of consistency between the cellular phenotype and the clinical findings in the patient [52]. Although the main strength of hiPSC-CMs is that they carry same genetic background as the donors, our experimental condition does not properly mimic the physiological condition since the experiments were conducted on two-dimensional cell culture [53, 54]. A three-dimensional culture environment could be is crucial not only to replicate an in vivo microenvironment, but also to improve the maturity of hiPSC-CMs [53, 54].

Finally, differences in gene expression and epigenetic signature might constitute additional phenotype-modifying factors. New information about disease modifiers is needed for the effective diagnosis and treatment of patients.

## Electronic supplementary material

Below is the link to the electronic supplementary material.

Additional File: SPIRIT 2013 Checklist: Recommended items to address in a clinical trial protocol and related documents

## Data Availability

All data generated or analysed during this study are included in this published article [and its supplementary information files].

## List of Abbreviations

AP: Action Potential
APA: Action Potential Amplitude
APD_50_: Action Potential Duration at 50% repolarization
APD_90_: Action Potential duration at 90% repolarization
BPM: Beat Per Minute
Ca^2+^: Calcium
cFPD: corrected Field Potential Duration
CMs: Cardiomyocytes
Vmax: Maximal upstroke velocity
EB: Embryoid Body
ECG: Electrocardiogram
FPD: Field Potential Duration
hiPSC: Human Induce Pluripotent Stem Cell
hiPSC-CMs: Human Induced Pluripotent Stem Cell derived Cardiomyocytes
I_Na_: Sodium Current
MDP: Maximum Diastolic Potential
MEA: Micro Electrode Array
Na^+^: Sodium
Na_V_1.5: Cardiac Sodium Channel
TA: Triggered Activity
V_1/2_: Half-maximum (in)activation Potential
k: Slope
τ_f_: Time constants of the fast-inactivating component
τ_s_: Time constants of the slow-inactivating components
