# Supplementary material for: HiPSC-derived cardiomyocyte to model Brugada syndrome: both asymptomatic and symptomatic mutation carriers reveal increased arrhythmogenicity
Source: BMC Cardiovasc Disord. 2023 Apr 25;23:208. doi: 10.1186/s12872-023-03234-7 (PMC10131315; doi:10.1186/s12872-023-03234-7)
Supplement: Supplementary file 1 — Additional File: SPIRIT 2013 Checklist: Recommended items to address in a clinical trial protocol and related documents [file 12872_2023_3234_MOESM1_ESM.docx]

**Supplementary Information**


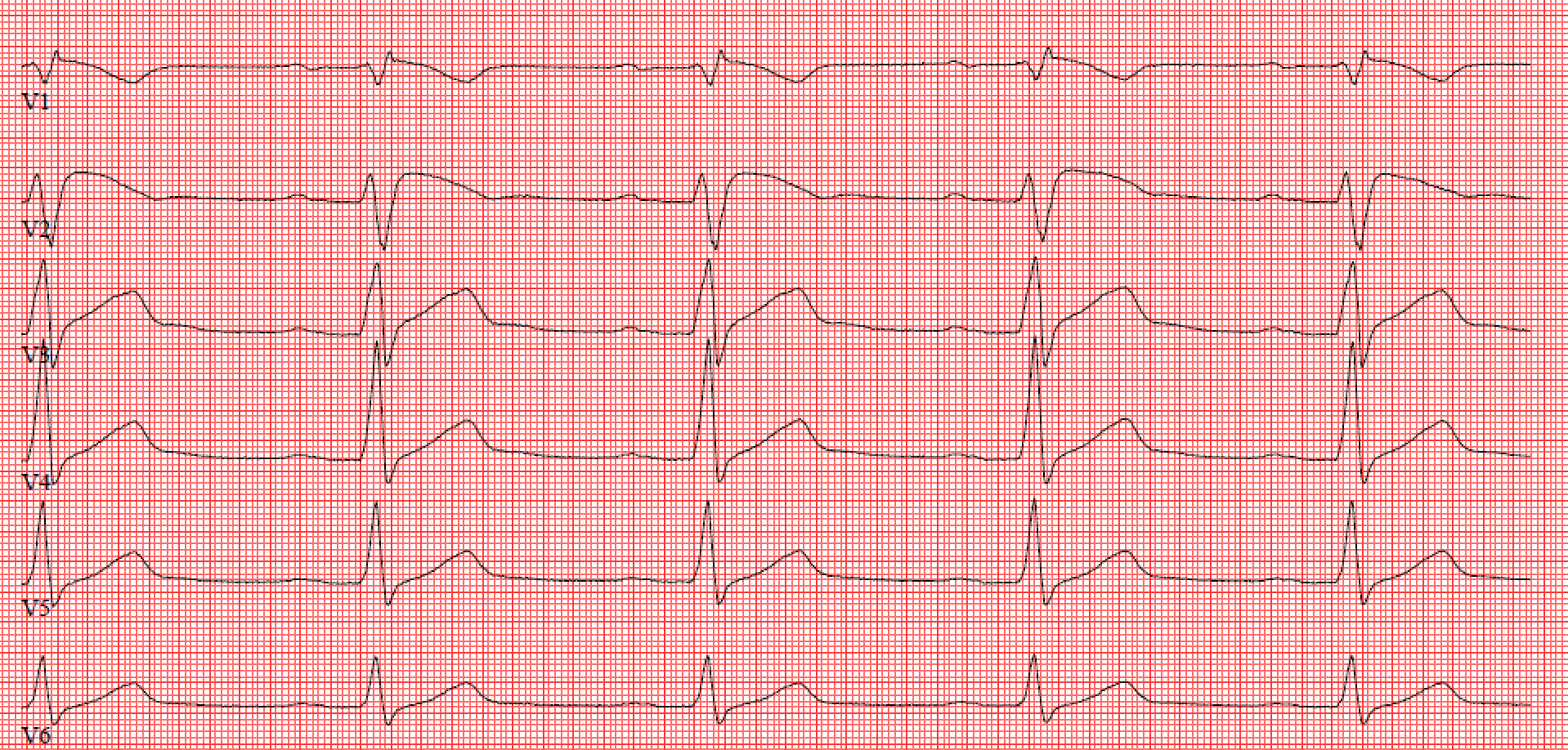


**Supplementary Figure 1:** Typical example of Brugada type 1 ECG obtained from the symptomatic individual after collapse.

**Supplementary Table 1.** Primers used for generating the plasmids.

| Assay | Primer/Probe | Sequence |
| --- | --- | --- |
| WT plasmid generation | Forward primer with BamHI restriction site  Reverse primer with  NotI restriction site | attaggatccgtcctgtctgactttgccgatg  taatgcggccgcgaagtcggcgagatcttcactgtg |
| Mutant plasmid generation | Mutagenesis sense  primer  Mutagenesis antisense  primer | tccagagagccttctgcaggcacctgctgca  tgcagcaggtgcctgcagaaggctctctgga |
| TaqMan SNP Genotyping  assay for allelic  imbalance determination | Forward primer  Reverse primer  Probe wild type VIC  Probe mutant FAM |  |

**

**

**Supplementary Figure 2:** Simultaneous recording of action potential (AP) and video recording. (A) Spontaneous APs recording in gap-free mode. (B) Synchronization pulses indicates the duration of video recording. (C) video traces corresponding to above APs. Indication of half-width contraction and relaxation duration.


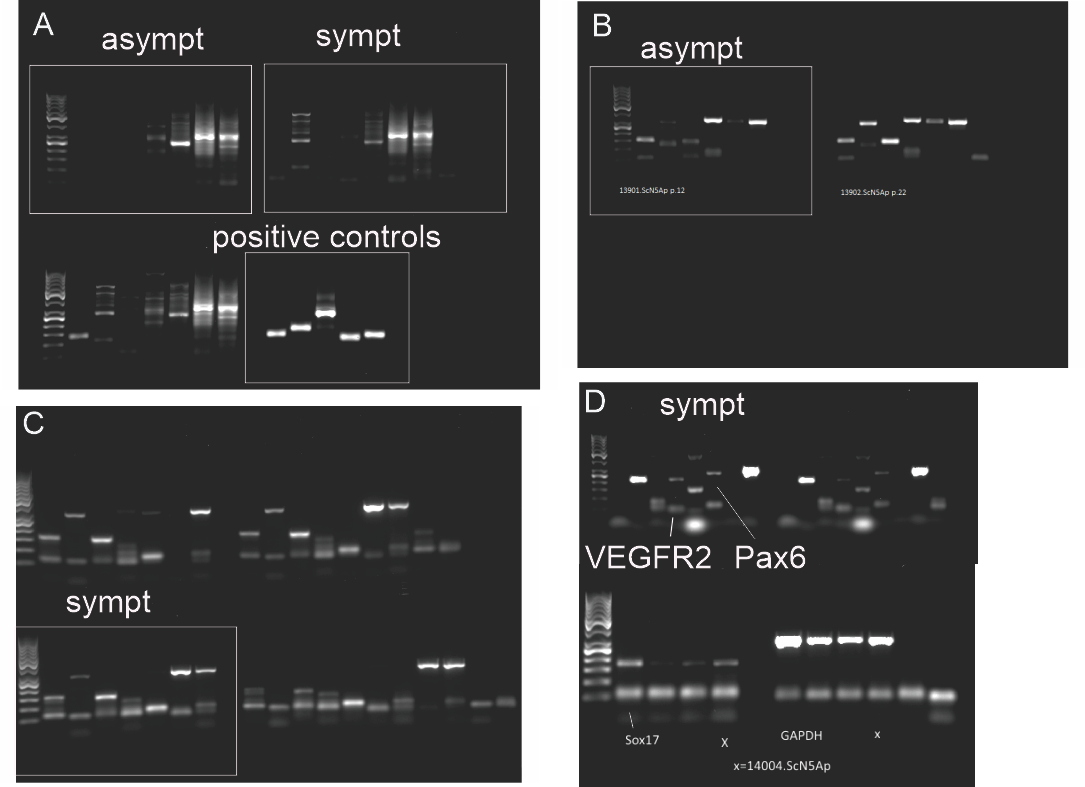


**Supplementary Figure 3:** iPSC charecterization full images. A) Exogenous genes are expressed in asymp-iPSC lines (Standard, Oct-3/4, Klf-4,Lin-28,Sox-2,L-Myc,GAPDH, b-actin), symp-iPSC lines (Standard, Oct-3/4, Klf-4,Lin-28,Sox-2,L-Myc,GAPDH, b-actin), and control (Oct-3/4, Klf-4,Lin-28,Sox-2,L-Myc,). B) Pluripotency markers are turned on in asympt-iPSC (Standard, Sox2, Nanog, OCT,c-myc,Rex1, GAPDH) C) Pluripotency markers are turned on in sympt-iPSC (Standard, Sox2, Nanog, OCT,c-myc,Rex1, GAPDH, B-actin) D) EBs express markers from all the three embryonic germ layers (VEGFR2, Pax6, Sox17, GAPDH).

**Supplementary Table 2.** Sodium current densities recorded from WT, asymptomatic and symptomatic CMs. mV represents the membrane potential. n represents the number of cells. Data are presented as mean±S.E.M. ns represents not statistically significant.

| **mV** | **WT (n=30)** | **asymt (n=21)** | **sympt (n=23)** | **Statistical significance** |
| --- | --- | --- | --- | --- |
| **-60** | -0.2±0.1 | -0.2±0.1 | -0.1±0.1 | ns |
| **-55** | -0.3±0.1 | -0.4±0.1 | -0.3±0.1 | ns |
| **-50** | -0.8±0.2 | -0.8±0.2 | -0.7±0.2 | ns |
| **-45** | -2.8±0.9 | -2.9±0.6 | -2.8±0.7 | ns |
| **-40** | -7.7±1.9 | -8.9±1.8 | -10.1±2.1 | ns |
| **-35** | -13.8±2.4 | -17.8±2.8 | -18.3±3.5 | ns |
| **-30** | -17.8±2.6 | -23.1±3.1 | -23.2±3.8 | ns |
| **-25** | -19.7±2.7 | -25.5±3.1 | -25.8±3.9 | ns |
| **-20** | -20.1±2.6 | -26.0±2.9 | -26.2±3.7 | ns |
| **-15** | -19.6±2.5 | -25.5±2.8 | -25.6±3.5 | ns |
| **-10** | -18.7±2.3 | -24.2±2.6 | -24.5±3.3 | ns |
| **-5** | -17.4±2.1 | -22.6±2.4 | -22.7±3.0 | ns |
| **0** | -16.0±2.0 | -20.9±2.2 | -20.8±2.7 | ns |
| **5** | -14.5±1.8 | -19.1±2.0 | -19.0±2.5 | ns |
| **10** | -13.1±1.6 | -17.3±1.8 | -17.1±2.2 | ns |
| **15** | -11.6±1.4 | -15.3±1.6 | -15.2±2.0 | ns |
| **20** | -10.3±1.3 | -13.7±1.4 | -13.3±1.8 | ns |
| **25** | -9.0±1.2 | -11.8±1.3 | -11.6±1.7 | ns |
| **30** | -7.7±1.0 | -9.8±1.3 | -9.9±1.5 | ns |
| **35** | -6.8±0.9 | -8.4±1.1 | -8.3±1.4 | ns |
| **40** | -5.7±0.8 | -6.8±1.0 | -7.0±1.2 | ns |
| **45** | -5.0±0.7 | -5.4±0.9 | -5.8±1.1 | ns |
| **50** | -4.0±0.7 | -4.3±0.9 | -4.6±1.0 | ns |
| **55** | -3.0±0.6 | -2.6±0.9 | -3.7±0.9 | ns |
| **60** | -2.5±0.5 | -1.1±1.0 | -2.7±0.9 | ns |
| **65** | -1.8±0.6 | 0.3±1.0 | -1.2±0.9 | ns |
| **70** | -1.2±0.6 | 2.0±1.1 | -0.2±0.9 | ns |
| **75** | -0.8±0.5 | 3.3±1.2 | 0.8±0.8 | ns |
| **80** | -0.2±0.7 | 3.8±1.5 | 6.7±4.5 | ns |

**Supplementary Table 3:** Voltage-dependence (in)activation for WT, asymptomatic and symptomatic CMs. n represents the number of cells. V_1/2_, voltage of half-maximal (in)activation and k, slope factor of voltage-dependence of (in)activation. Data are presented as mean±S.E.M. ns represents not statistically significant.

|  | **WT** | **asympt** | **sympt** | **Statistical significance** |
| --- | --- | --- | --- | --- |
| 1. **Voltage-dependence activation** | | | | |
|  | n=16 | n=19 | n=19 |  |
| **V_1/2_** | -31.0±1.1 | -33.5±1.0 | -31.9±1.7 | ns |
| **k** | 6.0±0.5 | 5.4±0.4 | 5.1±0.4 | ns |
| 1. **Voltage-dependence inactivation** | | | | |
|  | n=19 | n=19 | n=18 |  |
| **V_1/2_** | -71.5±2.0 | -73.1±1.2 | -74.1±1.5 | ns |
| **k** | 9.9±0.4 | 10.5±0.3 | 10.3±0.3 | ns |

**Supplementary Table 4:** Fast (τ_fast_) and slow time (τ_slow_) constant decay of sodium current over membrane potential (mV) calculated by fitting bi-exponential function for WT, asymptomatic and symptomatic CMs. n represents the number of cells. Data are presented as mean±S.E.M. * or # *P*<0.05, ** or ## *P*<0.01, *** or ### *P*<0.001 and ns, not statistically significant. * WT vs. asympt and # WT vs. sympt.

| **mV** | **WT (n=20)** | **asympt (n=17)** | **sympt (n=18)** | **Statistical significance** |
| --- | --- | --- | --- | --- |
|  | **τ_fast_** | | |  |
| -35 | 2.7±0.3 | 2.4±0.2 | 2.6±0.3 | ns |
| -30 | 2.4±0.3 | 1.9±0.1 | 1.9±0.1 | ns |
| -25 | 1.9±0.2 | 1.7±0.1 | 1.7±0.1 | ns |
| -20 | 2.0±0.2 | 1.4±0.1 | 1.5±0.1 | ns |
| -15 | 1.8±0.2 | 1.3±0.1 | 1.2±0.1 | # WT vs. sympt |
| -10 | 1.7±0.3 | 1.1±0.1 | 1.1±0.1 | * WT vs. asympt  # WT vs. sympt |
| -5 | 1.7±0.3 | 1.0±0.1 | 0.9±0.1 | * WT vs. asympt  ## WT vs. sympt |
| 0 | 1.4±0.2 | 1.0±0.1 | 0.8±0.1 | ## WT vs. sympt |
| 5 | 1.5±0.3 | 0.9±0.1 | 0.8±0.1 | * WT vs. asympt  ## WT vs. sympt |
| 10 | 1.3±0.2 | 1.0±0.1 | 0.7±0.1 | ### WT vs. sympt |
| 15 | 1.0±0.1 | 1.0±0.1 | 0.9±0.1 | ns |
| 20 | 1.0±0.1 | 0.9±0.1 | 0.8±0.1 | ns |
|  | **τ_slow_** | | |  |
| -35 | 18.2±2.6 | 17.8±1.8 | 17.2±1.6 | ns |
| -30 | 14.9±2.4 | 12.6±1.5 | 13.9±1.9 | ns |
| -25 | 13.7±2.1 | 11.1±2.0 | 10.8±1.5 | ns |
| -20 | 10.0±1.5 | 7.3±0.5 | 8.6±0.8 | ns |
| -15 | 8.1±0.5 | 7.6±0.5 | 7.4±0.6 | ns |
| -10 | 8.1±0.6 | 7.1±0.4 | 7.9±0.9 | ns |
| -5 | 7.4±0.5 | 7.2±0.3 | 6,8±0,3 | ns |
| 0 | 8.0±0.6 | 7.7±0.6 | 8.4±0.7 | ns |
| 5 | 9.2±0.9 | 7.8±0.4 | 8.0±0.4 | ns |
| 10 | 8.6±0.7 | 8.7±0.5 | 8.9±0.6 | ns |
| 15 | 9.0±0.7 | 9.3±0.7 | 9.5±0.7 | ns |
| 20 | 12.8±2.5 | 10.5±0.6 | 10.6±1.0 | ns |

**Supplementary Table 5.** Time course of recovery from inactivation and time-course of entry into the slow inactivation state for WT, asymptomatic and symptomatic CMs. Fast (τ_fast_) and slow time (τ_slow_) constant decay were calculated by fitting bi-exponential function. n represents the number of cells used. Data are presented as mean±S.E.M. * *P*<0.05, ** *P*<0.01, and ns, not statistically significant. * WT vs. asympt.

|  | **WT** | **asympt** | **sympt** | **Statistical significance** |
| --- | --- | --- | --- | --- |
| **A** | **Time-course of recovery after inactivation** | | |  |
|  | n=7 | n=8 | n=8 |  |
| **τ_fast_** | 74.8±7.0 | 47.4±4.9 | 63.3±5.7 | * WT vs. asympt |
| **τ_slow_** | 700.3±94.1 | 509.2±60.5 | 579.9±32.3 | ns |
| **B** | **The time-course of entry into the slow inactivation state** | | |  |
|  | n=9 | n=6 | n=9 |  |
| **τ_fast_** | 5.9±0.7 | 11.9±2.1 | 10.1±1.5 | * WT vs. asympt |
| **τ_slow_** | 187.6±40.5 | 422.6±53.5 | 252.0±23.4 | ** WT vs. asympt |





**Supplementary Figure 4. I_Kr_ and Calcium current densities.** **A-B.** Current-voltage relationships of average I_Kr_ densities. from WT, asymptomatic and symptomatic CMs. **C.** Current-voltage relationships of average I_Ca,L_ densities. **E.** Average voltage dependence of activation. **D** Time-course of recovery after inactivation. Peak I_Ca,L_ elicited by P2 were normalized (P2/P1) and plotted as function of the recovery interval. **E.** The time-course of entry into the slow inactivation state. Peak I_Ca,L_ elicited by P2 were normalized (P2/P1) and plotted as function of the duration of P1. **F-G.** Average fast (τ_fast_) and slow (τ_slow_) time constants from recovery after inactivation and entry into the slow inactivation state. Insets: Voltage-clamp protocol used in each experiment. Data are presented as mean±S.E.M.

**Supplementary Table 6.** Half-width contraction and relaxation duration calculated from video recording of WT, asymptomatic and symptomatic CMs. n represents the number of cells used. Data are presented as mean±S.E.M, ns, not statistically significant.

|  | **WT (n=27)** | **asympt (n=20)** | **sympt (n=23)** | **Statistical significance** |
| --- | --- | --- | --- | --- |
| **Contraction duration (ms)** | 106.1±9.4 | 82.3±6.9 | 78.3±4.8 | ns |
| **Relaxation duration (ms)** | 118.3±9.2 | 97.6±5.5 | 93.8±5.4 | ns |

**Supplementary Table 7.** MEA recordings from WT, asymptomatic and symptomatic CMs. The field potential duration (FPD) is corrected for beat rate to get corrected FPD (cFPD). n represents the number of cells used. Data are presented as mean±S.E.M, ns, not statistically significant.

|  | **WT**  **(n=13)** | **asympt (n=6)** | **sympt (n=19)** | **Statistical significance** |
| --- | --- | --- | --- | --- |
| **Beat rate (bpm)** | 49.1±2.9 | 50.8±4.4 | 47.0±3.9 | ns |
| **FPD (ms)** | 839.4±44.1 | 729.6±52.0 | 930.2±96.5 | ns |
| **cFPD (ms)** | 743.6±21.7 | 659.4±32.1 | 755.2±42.5 | ns |

|  | **Baseline** | | | | **Adrenaline** | | | |
| --- | --- | --- | --- | --- | --- | --- | --- | --- |
|  |  |  |  | **Statistical significance** |  |  |  | **Statistical significance** |
|  | **WT**  **n=58** | **asympt**  **n=95** | **sympt**  **n=165** |  | **WT**  **n=58** | **asympt**  **n=95** | **sympt**  **n=165** |  |
| **F/F0** | 2.42±0.16 | 2.07±0.23 | 1.81±0.14 | *** WT vs. sympt  ** WT vs. asympt | 1.88±0.13### | 1.82±0.20### | 1.69±0.13# | * WT vs Symp |
| **Ca^2+^ peak duration (ms)** | 1741.96±114.63 | 1206.03±78.51 | 1338.58±64.69 | ** WT vs. sympt  *** WT vs. asympt | 1406.10±95.00### | 1035.64±106.50### | 1087.82±51.18### | ** WT vs. sympt  *** WT vs. asympt |
| **Half-width (ms)** | 764.22±55.44 | 491.97±39.14 | 599.77±37.81 | ** WT vs. sympt  *** WT vs. asympt | 636.13±45.17### | 451.37±35.13## | 526.02±26.41## | ** WT vs. sympt  *** WT vs. asympt |
| **Decay time (ms)** | 1017.52±69.42 | 669.72±53.95 | 783.05±44.92 | ** WT vs. sympt  *** WT vs. asympt | 845.84±63.02### | 599.05±114.22### | 566.29±25.76### | *** WT vs. sympt  *** WT vs. asympt |
| **Interevent interval (ms)** | 3251.36±263.27 | 1967.63±131.16 | 2499.83±148.10 | ** WT vs. sympt  *** WT vs. asympt | 2352.59±173.42### | 1347.81±75.59### | 1736.78±89.03### | * sympt vs. asympt  ** WT vs. sympt  *** WT vs. asympt |
| **Event frequency (Hz)** | 0.42±0.04 | 0.74±0.05 | 0.62±0.03 | * sympt vs. asympt  ** WT vs. sympt  *** WT vs. asympt | 0.58±0.04### | 0.99±0.06### | 0.81±0.04### | * sympt vs. asympt  ** WT vs. sympt  *** WT vs. asympt |

**Supplementary Table 8.** Comparisons of the amplitude, Ca^2+^ peak duration (Ca^90^), half-width, decay time 90% to 10%, interevent interval and event frequency of WT, asymptomatic and symptomatic CMs during adrenaline perfusion. Data are presented as mean±S.E.M. * or # indicates *P*<0.05, ** or ## *P*<0.01 and *** or ### *P*<0.001. * indicates differences between cell lines and # indicates differences inside cell line between baseline and adrenaline.

**Supplementary Table 9.** Averages and SD of Ca^2+^ peak parameters of baseline and after flecainide perfusion. # indicates significant differences inside cell line between baseline and flecainide, # *P*<0.05, ## *P*<0.01, ###, *P*<0.001. No statistically significant differences in Ca^2+^ peak parameters were seen between WT, asymptomatic and symptomatic CMs during flecainide perfusion.

|  | **Baseline** | | | | **Flecainide** | | | |
| --- | --- | --- | --- | --- | --- | --- | --- | --- |
|  |  |  |  | **Statistical significance** |  |  |  | **Statistical significance** |
|  | **WT**  **n=97** | **asympt**  **n=48** | **sympt**  **n=54** |  | **WT**  **n=97** | **asympt**  **n=48** | **sympt**  **n=54** |  |
| **F/F0** | 1.74±0.13 | 1.75±0.16 | 1.83±0.12 | ns | 1.44±0.10### | 1.59±0.15# | 1.57±0.12## | ns |
| **Duration (ms)** | 1334.2±102.6 | 1177.0±99.6 | 1292.3±83.7 | ns | 1683.5±132.4### | 1220.2±82.1 | 1402.2±108.9# | ns |
| **Half-width (ms)** | 568.0±40.8 | 484.0±36.6 | 600.5±44.1 | ns | 750.7±78.1### | 514.7±36.8# | 710.6±60.9### | ns |
| **Interevent interval (ms)** | 2667.3±215.2 | 1960.4±179.9 | 2726.1±249.8 | ns | 3365.0±285.6### | 2436.3±200.3### | 3193.1±297.0## | ns |
| **Event frequency (Hz)** | 0.62±0.04 | 0.66±0.05 | 0.52±0.04 | ns | 0.49±0.03### | 0.55±0.04### | 0.47±0.04## | ns |
